# Using the Edinburgh Visual Gait Score to Compare Ankle-Foot Orthoses, Sensorimotor Orthoses and Barefoot Gait Pattern in Children with Cerebral Palsy

**DOI:** 10.3390/children7060054

**Published:** 2020-06-01

**Authors:** Clare MacFarlane, Wayne Hing, Robin Orr

**Affiliations:** Faculty of Health Sciences and Medicine, Bond University, Gold Coast, QLD 4226, Australia; whing@bond.edu.au (W.H.); rorr@bond.edu.au (R.O.)

**Keywords:** ankle-foot orthoses, sensorimotor orthoses, gait, Edinburgh Visual Gait Score, cerebral palsy, children

## Abstract

Gait analysis is one aspect of evaluation in ambulatory children with cerebral palsy (CP). Ankle-foot orthoses (AFOs) improve gait and alignment through providing support. An alternative and under-researched orthosis are sensomotoric orthoses (SMotOs). The Edinburgh Visual Gait Score (EVGS) is a valid observational gait analysis scale to measure gait quality. The aim of this study was to use the EVGS to determine what effect AFOs and SMotOs have on gait in children with CP. The inclusion criteria were: mobilizing children with a CP diagnosis, no surgery in the past six weeks, and currently using SMotOs and AFOs. Eleven participants were videoed walking 5 m (any order) barefoot, in SMotOs and AFOs. Of the participants (age range 3–13 years, mean 5.5 ± 2.9), two were female and six used assistive devices. Seven could walk barefoot. Participants had spastic diplegia (4), spastic quadriplegia (6), and spastic dystonic quadriplegia (1). Gross Motor Functional Classification System (GMFCS) levels ranged I–IV. The total score for SMotOs (7.62) and AFOs (14.18) demonstrated improved gait when wearing SMotOs (no significant differences between barefoot and AFOs). SMotOs may be a viable option to improve gait in this population. Additional study is required but SMotOs may be useful in clinical settings.

## 1. Introduction

Cerebral palsy (CP) is a neurodevelopmental condition well recognized to begin at birth or early childhood and persist through the lifespan [1]. It has been defined as a group of permanent disorders of the development of movement and postures, causing activity limitation through spasticity [2,3], muscle weakness, impaired postural control, and selective motor control as some of the primary manifestations of this brain injury [3]. CP is often accompanied by disturbances of sensation, perception, cognition, communication, behavior, and by epilepsy. One of these activity limitations may be the ability and co-ordination for walking (gait), with control of movements and postures being affected. As ambulation is the usual method for mobilizing, many children with CP strive to achieve any form of walking possible, whether it is with or without an assistive device.

Gait assessment assists in determining the degree and cause of gait abnormality and can be used as an outcome measure to evaluate change and the effectiveness of an intervention [4,5,6]. Instrumented gait analysis is the gold standard for the evaluation of movement [4] but requires highly technological equipment in a specialised gait laboratory. A gait laboratory requires considerable capital investment, trained personnel, and is often not readily accessible for routine clinical work [6,7]. Observational gait assessment is considered as a cost effective alternate for instrumented gait analysis in regular clinical practice [4]. One observational tool used in clinical settings is the Edinburgh Visual Gait Score (EVGS). The EVGS has been demonstrated as a valid and reliable [8,9], clinically applicable visual gait analyses tool for children with CP [9].

Ankle-foot orthotics (AFOs) are the typical prescription of lower extremity orthoses for the management of lower limb deformities that often occur with CP. A consensus conference of the International Society for Prosthetics and Orthotics identified the aims of lower extremity orthotic management in children with CP: (1) to correct and/or prevent deformity, (2) provide a base of support, (3) facilitate training of motor skills, and (4) improve efficiency of walking [10]. The goal of each AFO prescribed for a child with CP is the collective improvement of these biomechanical variables to increase the ease of taking an individual step with the potential to enhance walking activity and functional skills [11]. AFOs are designed to; affect body structure [3], support normal joint alignment and mechanics, provide variable range of motion (ROM) when appropriate, facilitate function [3,12,13,14], stabilize the ankle/foot complex [15], and enable a continuous Achilles/gastrocnemius stretch [16,17,18]. Along with joint alignment, other improvements that may be seen through the use of AFOs are an improvement in walking efficiency [10,19], position of the foot for function, [20], and improvement in gait function and pain prevention [21].

There are multiple studies assessing and comparing the motor changes in gait and other gross motor skills using different types of AFOs in children with CP [15,22,23]. Buckon et al. [15], noted that the AFOs did not significantly improve standing skills of the GMFM-88 (*p* ≤ 0.025), but reported an improvement of the walking, running, and jumping elements when wearing AFOs (*p* < 0.002) compared to barefoot. In another study, Buckon et al. [22] noted that despite the AFO enhancing stability throughout static (e.g., standing) and dynamic (e.g., walking) gross motor and functional skills, they did not allow the child to achieve a skill they were previously unable to master. Dalvand et al. [23] also noted improvements in gait with both solid-AFO and hinged-AFO, although hinged-AFO demonstrated better improvements in gross motor function than solid-AFO. 

Unlike AFOs, sensomotoric orthotics (SMotO) provide a different approach to the management of gait in children with CP. Wegner et al. [24] describe one adaptation theory as ‘elements’ on the foot orthoses (e.g., forefoot valgus posting or lateral rearfoot padding) increase local pressures which are detected by cutaneous receptors, muscle spindles or the Golgi apparatus. To expand this, SMotOs are created to directly change the muscle length [25] and ”activate and deactivate” muscles by increasing or decreasing individually placed point specific pressure on musculotendinous structures in the foot of the tibialis posterior, peroneus brevis, and the lumbricals/quadratus plantae. This theory implies the information that is transmitted by the sensors for the control of muscle activity is changed [26]. Depending on these individual pressure bumps’ height and placement, the muscles can be activated or restricted [25,26]. CP affects the different areas of the brain, therefore interrupting signals sent to the muscles. The SMotOs work on the idea that the signals are being sent from the muscles back up to the spinal cord through activation of the Golgi bodies, therefore signaling muscles to respond to stimulus. Clinically, the SMotO have demonstrated improved foot alignment, balance, control with walking, and functional skills. Clients have been prescribed the SMotOs as a supplementary lower limb orthosis when it is noted they are finding functional movement restricted in the AFO. SMotOs have also been prescribed for children who require more feedback from their feet, where wearing shoes alone has not been effective.

There is a lack of evidence examining the ‘sensorimotor response’ paradigm, as there, there are no randomized trials, minimal peer-reviewed papers in English, and only a few small cross-sectional pediatric papers [24]. There is only one paper in English reporting the use of SMotOs on in-toeing gait in children (with idiopathic in-toeing or clubfoot) and it found that they improved abnormal gait patterns of pediatric in-toeing gait by decreasing femoral internal rotation through the end of the swing phase and the beginning of the stance phase and by decreasing tibial internal rotation during the stance phase [27]. 

There are numerous papers that demonstrate improved gait when wearing AFOS, but there have been no studies to date comparing the effect of SMotOs and AFOs on gait in children with CP. Clinically, there appears to be improvements in gait quality when children with CP wear SMotOs. Therefore, this study aims to compare the changes in gait from barefoot when children with CP are wearing SMotOs and AFOs, through use of the EVGS.

## 2. Experimental Section

Ethical approval was obtained through the Bond University Research and Ethics Committee (Approval RO-1835). Consent was gained from clinic directors in both private practice settings. Participants and their caregivers were given an explanatory statement and consent form both of which were read and completed before data collection took place.

The participant inclusion criteria were: (a) child with a diagnosis of CP, (b) no surgery in past six weeks, (c) currently using SMotO and AFOs (completed the wearing in process of at least two weeks), and (d) able to mobilize (with or without a device). Participants were recruited by convenience sampling and were assessed through two private pediatric therapy practices in Sydney (Therapies for Kids and NAPA Centre). There was no limit on participation due to GMFCS level. Participants brought their own SMotOs and AFOs to the data collection sessions.

**Figure 1:**
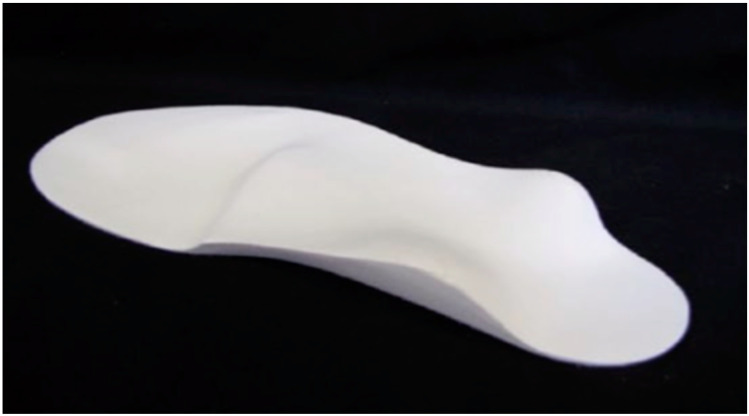
Sensorimotor orthosis [24].

**Figure 2:**
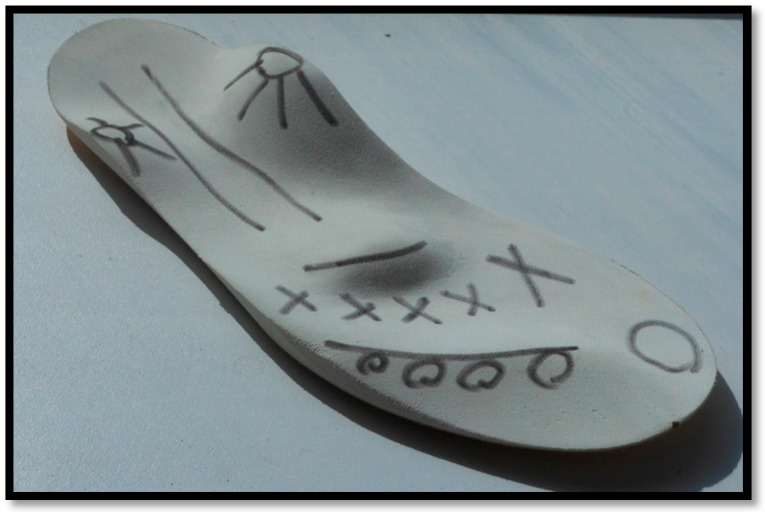
Sensorimotor orthosis with descriptive markings.

The AFOs were custom-made through public or private orthotists from polypropylene with velcro straps holding the foot in place. The AFOs all had a full-length foot plate. Due to the AFOs being the participants ‘usual prescription’ there was no further assessment or measurements of the AFOs performed. The SMotOs were custom made for each child from ethyl vinyl acetate and had been assessed and prescribed by a podiatrist or pedorthist who were both expert in this design type of orthosis. Figure 1 shows the finished SMotO [24]. Figure 2 illustrates the placement of toes (circles) metatarsal heads (crosses), and plantar fascia (lines).

The Golgi bodies (in the musculotendinous junctions of the tendons of the foot) are activated to switch the muscle ‘on’ or ‘off’ by the pressure from the ‘bumps’ coinciding with the musculotendinous junctions [28] (Figure 2). It has been proposed that information is sent by afferent feedback pathways (centrally) in order to reduce the activity of over-active muscles through inhibition, which in turn facilitates an increase in the activity of weaker muscles [29]. Given the physiology of ascending muscle chains, the reaction will not only affect the single muscle targeted (e.g., in the foot) but influence the complete chain of movement (e.g., ankle and lower limb) and positively impact malposition [26] (e.g., over pronation).

The AFOs and SMotOs were worn inside the participants usual shoe (for that particular orthoses). Participants were asked to walk barefoot (where appropriate, due to ability to mobilize without any type of foot support), in SMotOs, and in AFOs for at least 5 m at a self-directed pace. To provide motivation, participants were able to choose the order in which they wore the orthoses. Video imagery was taken with a handheld device (Apple iPhone 7s, Apple Inc., CA, USA) in anterior, posterior and lateral views. Videos were taken in a well-lit environment, following for lateral views or zooming in and out (as needed) when the child walked. If required due to poor video quality, misbehaviour, or misstep, additional videos were taken to ensure a quality video was assessed. The child was allowed the comfort of a break between walks if needed. To ensure validity and reliability of video, the plane of motion was followed.

To determine changes in gait, the EVGS was used. The EVGS comprises 17 parameters for each lower limb and evaluates movement across six sites (trunk, pelvis, hip, knee, ankle, and foot) [9]. Each gait phase is analyzed in the frontal, sagittal, and transverse planes and the anatomical sites are evaluated for movement through video observation [30]. Scoring uses a 3-point ordinal scale. When the segment is marked 0, it determines a normal score. When there is a 1, it means a moderate deviation from normal in either direction, and 2 relates to a marked deviation, therefore a higher score relates to a more severe deviation or abnormality of gait. The developers of EVGS reported a score reduction of 4 on each limb (compared to pre-intervention score) as an improvement and as the minimum change in score required that would be indicative of change, not merely related to observer variation [9].

The EVGS is a valid, robust, reliable, and easy-to-use observational gait analysis scale to measure gait quality in children with CP [4,9,31]. It has been examined for the purpose of orthosis evaluation in adults [32] but not yet validated in children with orthoses. The scale has stringent instructions to ensure reliability. Its agreement and validity with three-dimensional gait analysis have been documented [8] and was noted to be 52%–73%. The essential properties of an observation scale are validity, reliability, and ability to detect change [33]. Responsiveness is the ability of a tool’s detection of change due to an intervention or over time. The EVGS is shown to correlate with the Gait Profile Score and the GMFCS [34], two relevant and valid measures relating to CP. Frame by frame analysis was performed to score the gait using the EVGS with all analysis performed by the principal researcher, a physiotherapist who works with the children and has 6 years of experience using this tool. This analysis took place after all face-to-face data collection had been completed, thereby minimizing time pressures on the families and their child. As the participant walked 5 m for each camera direction, there was more than one stride to observe. Once viewed, the most usual score was used.

### Data Analysis

The EVGS was analyzed through SPSS statistics software (Version 20.0, IBM, NY, USA) and the Microsoft Office Excel 2007 (Microsoft, WA, USA) were used for the data entry and analysis. Normality was determined via visual inspection of histograms, box plots and normal Q-Q plots. Depending on distribution, parametric or non-parametric tests were used to determine if there were any significant differences in the baseline characteristics or of the groups. Descriptive statistics were used to profile the data: the median difference of the total EVGS scores and the mean difference of the average walking score, before and after the intervention, were calculated. A summated score of each limb was used for data analysis for this study. Thus, the score for the EVGS ranges from 0 to 34 on left (L) or right (R). The data for the barefoot condition and both orthoses were then statistically analyzed through a one-way ANOVA to determine significance and post hoc Bonferroni to outline further comparison significance. There was also a cumulative total of each segment analysed. Repeated measures of ANOVA with Bonferroni post hoc and Wilk’s Λ was performed. Alpha levels were set at 0.05 a priori.

## 3. Results

### 3.1. Participants

From 27 possible participants, 10 were unable to participate due to inability to understand instruction or poor comprehension, and four were unable to attend data collection. From the 13 potential participants, two were excluded as they did not wear AFOs anymore. See Table 1 for full participant demographics.

Of the final yield of 11 participants (aged between 3–13 years with average 5.5 ± 2.9 years), seven were able to walk barefoot and therefore had barefoot data collection recorded. Four were unable to walk barefoot due to inability or child refusal. There were four participants with spastic diplegia, six with spastic quadriplegia and one with spastic dystonic quadriplegia. The GMFCS levels of participants were: one level I, four level II, three level III, three level IV. Six participants used assistive devices for EVGS (participants 1, 2, 8, and 11 used a reverse walker, participant 6 used a Rifton pacer, and participant 10 used a Buddy Roamer). Five participants wore hinged-AFOs (participants 3, 4, 6, 7, and 11) and the remaining participants wore solid-AFOs. One participant had ethyl vinyl acetate (EVA) heel wedges on their solid-AFO to encourage weight through the heel, mimicking heel strike. There were no data recorded of orthosis use timing per child over usual day due to this research focusing on the immediate effect of orthoses on gait.

### 3.2. Scores

The total EVGS for (L) and (R) barefoot (where applicable) and with each orthosis are described in Table 2. See Appendix A for full extrapolated data set tables for each participant and segmental totals.

When barefoot was assessed, the overall scores across participants demonstrated a poorer score than both AFO and SMotO, except for one participant who demonstrated poorer results when wearing AFOs compared to barefoot and SMotO. The descriptive statistics of EVGS are provided in Table 3 outlining the mean and SD for total (L) and (R) scores for barefoot, AFO, and SMotO intervention.

One-way ANOVA analyses revealed significant differences between total (L) (*p* = 0.011) and (R) (*p* = 0.014) scores between SMotO and AFOs (Table 4).

There were significant differences on the (L) lower limb between barefoot and SMotO (*p* = 0.032), and AFO and SMotO (*p* = 0.027). On the (R) lower limb, there were significant differences between AFO and SMotO (*p* = 0.028).

In the segmental analyses, repeated measures ANOVA elicited statistically significant differences in the foot, F (2, 5) = 8.993, *p* < 0.022; Wilk’s Λ = 0.218, partial η2 = 0.782, and hip, F (2,5) = 6.10, *p* < 0.045; Wilk’s Λ = 0.290, partial η2 = 0.710, with the biggest effect in the foot. Post hoc analysis with a Bonferroni adjustment revealed statistically significant differences between barefoot and SMotOs in the foot, mean difference (MD) = 8.86 (95% confidence interval [CI] 2.38 to 15.33, *p* = 0.012), and between AFO and SMotOs at the hip, MD = 1.14 (95% CI, 0.03 to 2.26, *p* = 0.046).

## 4. Discussion

The aim of this study was to investigate the changes two types of orthoses (SMotOs and AFOs) had on gait pattern in children with CP, as derived through the EVGS. All the participants were in GMFCS levels I–IV and used orthoses to walk, both in SMotOs and AFOs. There were six participants who required the use of ambulatory aids, displaying a varied range of gait ability. Overall, this cross-sectional cohort study found SMotOs to have a more positive influence on gait pattern compared to AFO and barefoot.

The total raw scores of each participant demonstrate that more desirable gait patterns were observed when wearing SMotO. This was resultant across 11 participants by a lower total score when wearing SMotOs on both the left (7.46) and right (7.77) compared to when wearing AFOs on the left (14.00) and right (14.36) and the seven participants with barefoot left (14.22) and right (14.11). Due to limitations in assessing calcaneal alignment in orthoses, the EVGS results would be affected at the foot / ankle when using restrictive orthoses compared to more dynamic orthoses that don’t limit ankle movement. Barring participants 3 and 5, the general trend indicates that SMotO had the lower scores, which correlates to the EVGS score line indicating a more aligned gait pattern. The one-way ANOVA confirms that there was a significant difference in EVGS scores between the use of SMotO and AFO. One participant demonstrated a worse score on the (L) foot when wearing SMotO compared to AFO due to poor foot and knee alignment. Subsequently, a significant difference on the left lower limb was found when participants wore SMotOs compared to barefoot or AFOs, but not the right lower limb. The right lower extremity score was close to being significant (*p* = 0.052) when comparing SMotO to barefoot or AFOs but may need a larger yield study to determine its significance and if it is a usual trend. Interestingly, there was no significant difference between the AFO and barefoot scores.

Looking at the segmental breakdown of the EVGS, the hip and foot are seen to be most affected by orthotic intervention. It is found that that in the foot, barefoot is significantly different from SMotO (*p* = 0.012) and in the hip there is a significant difference between the AFO and SMotO (*p* = 0.046). The differences in the foot results between barefoot and SMotO may demonstrate the theory presented earlier by Wegner et al. [24]. Interestingly, there is no difference noted at the pelvis between barefoot and AFO. At the trunk, the AFOs presented a higher score than either barefoot or SMotOs, but this was not significant.

With regard to using the EVGS as an outcome measure to assess the effect of orthoses, a search for papers assessing the effect of AFOs on gait in children with CP through the EVGS only resulted in one case study paper by Young and Jackson [35]. This paper followed a child with spastic bilateral CP over 15 months, in which she was prescribed AFOs and began to stand and walk independently. Post AFO prescription, they noted clinically significant differences in the EVGS (increase by 7 points on the left and 11 points on the right—MCID of 2.4) and gait speed (42.9% increase in speed—MCID >10.9% is noted as large). This paper noted that AFOs did create a significant improvement in gait compared to barefoot. The lack of multiple papers using the EVGS to compare the effects of AFOs on gait in children with CP may provide a direction for future research.

One of the most common gait anomalies found in children with CP was in-toeing (amongst others) [36]. Although in a different population, there was one paper supporting the use of SMotOs to correct in-toeing in children with idiopathic in-toeing gait or clubfoot. Mabuchi [27] assessed the biomechanical effect of these orthoses on in-toeing gait in children. They found that the orthoses showed significant decreases in internal rotation at the proximal femur (loading response phase −18.3° ± 28.1° versus −21.6° ± 28.0°, *P* = 0.009 and terminal swing phase −16.3° ± 27.4° versus −19.0° ± 26.4°, *P* = 0.047) and the tibia in mid stance phase (0.7° ± 12.5° versus −2.0°± 14.9°, *P* = 0.030) and terminal stance phase (1.4° ± 11.9° versus −2.3° ± 14.5°, *P* = 0.042). They also found a significant increase in walking speed (67.9 m/min versus 64.9 m/min, *P <* 0.001) and stride length (500 mm versus 477 mm, *P <* 0.001). This may provide a basis to address in-toeing in children with CP with SMotOs.

The small, heterogenous sample affects the strength and generalizability of the results. Therefore, it is recommended that future research includes larger, more homogenous samples investigating SMotOs as another form of orthotic therapy for children with varied types of CP. It may also create opportunities to further investigate clinically useful observational gait assessment tools, such as the EVGS, for outcome measures when prescribing interventions such as orthoses. In support of Jagademma et al. [37], who stated that when investigating the effects of interventions such as AFOs, it is important to categorise children with CP based on their gait abnormalities. Therefore, it may be beneficial to further investigate multiple orthoses options or combinations than just AFOs alone, depending on the child’s needs. Future research could include validating the EVGS as clinical assessment tool for use in children with lower limb orthoses, comparing customised, tuned AFOs with SMotOs in three-dimensional gait analysis, or expanding this study by removing limitations and performing over a longer time period.

Limitations of the Study: Due to the researchers only being able to draw participants from the population we had access to, there were limitations on the number of potential participants. Limitations to this research include a small final yield of participants available to collect the full range of data, mainly due to the restrictions uncovered such as comprehension of the task, ability to follow direction, and ability to attend data collection. This may affect the strength of the results, although papers within the literature demonstrate a range of participant numbers whilst using the EVGS from 7 [30] to 151 [31]. With a longer recruitment period and implementation of this as ‘usual clinical practice’ in prescription of SMotOs, a larger group could be assessed for continuation of these research findings. Another limitation was the lack of tuning the AFOs to avoid the possibility of iatrogenic gait compensations. Tuning of AFOs is recommended by Owen [38] and Eddison & Chockalingam [39] but was not performed in this setting as the researchers were investigating the usual prescription of AFOs and SMotOs on gait pattern. The angle in the ankle and knee in AFOs were not assessed for angle during this study which may provide a limitation to the strength of this study. Ideally, this study would have provided customised AFOs with in-depth explanation of prescription process. This would thus enhance the quality of study and reduce possible suboptimal AFO prescription. Unfortunately, this process is complex, can be expensive and was not possible at this stage. Potential bias is another limitation acknowledged in this paper and would be better resolved with EVGS completed by blinded raters with experience as well as more stringent patient preparation and video capture methodology. Along with this, the EVGS has not been assessed for reliability and validity when observing children in orthoses, possibly due to the inability to observe the calcaneus in orthoses or shoes. Ong, Hillman & Robb [40] validated the EVGS’ reliability and validity for experienced observers in gait analysis. They noted, however, that the inexperienced observers were less accurate, and the experienced observers demonstrated more accurate results when compared to three-dimensional gait analysis. Three-dimensional gait analysis would be the preferred method of assessment for this type of study, but access to such a system was not possible. The EVGS does not allow for specific reporting of deviation direction, but rather indicated a deviation from ‘normal’, which may not be enough detail for some gait analyses.

## 5. Conclusions

The results of this study suggest that further evaluation of the effects of SMotO are warranted but the SMotO may, clinically, be an effective orthosis intervention to improve gait in children with CP. These results would be better validated if further research is performed in a gait laboratory using the gold standard three-dimensional gait analysis versus an observational score as there are many aspects of gait analysis. These results encourage further investigation into the use of SMotO in children with CP or to further specify the areas of benefit of the SMotO alongside AFO in relation to this population, their gait function and level of disability. Clinically, this creates an alternate orthoses prescription possibility for children with CP.

## Figures and Tables

**Table 1:** Participant demographics.

Participant	CP Type	GMFCS Level	Age (whole Years)	Walking Aid
1	Sp Dip	III	3	Reverse walker
2	Sp Q	III	7	Reverse walker
3	Sp Dip	I	4	
4	Sp Dip	II	4	
5	Sp Q	IV	3	Rifton Pacer
6	Sp Q	II	6	
7	Sp Q	II	13	
8	Sp Dip	III	3	Reverse walker
9	Sp Dys Q	IV	4	Buddy Roamer
10	Sp Q	IV	6	Reverse walker
11	Sp Q	II	8	

CP = cerebral palsy; Sp Dip = spastic diplegia; Sp Q = spastic quadriplegia; Sp Dys Q = spastic dystonic quadriplegia; GMFCS = Gross Motor Function Classification System.

**Table 2:** Total (L) and (R) Edinburgh Visual Gait Score (EVGS) for participants.

Participant	GMFCS Level	Total score barefoot		Total score AFO		Total score SMotO	
		(L)	(R)	(L)	(R)	(L)	(R)
**1**	III	26	27	20	21	14	16
2	III	11	13	6	9	4	4
3	I	20	16	12	12	17	10
4	II	11	15	16	15	7	8
5	IV	13	9	12	11	8	8
6	II	25	26	14	16	7	10
7	II	12	14	8	9	4	5
8	III	-	-	18	20	11	14
9	IV	-	-	20	20	12	12
10	IV	-	-	14	14	6	6
11	II	-	-	14	11	4	5

GMFCS = Gross Motor Functional Classification Scale; (L) = left; (R) = right; AFO = ankle-foot orthoses; SMotO = sensomotoric orthoses; - = no data due to inability or refusal to walk barefoot.

**Table 3:** Data descriptors for total (L) and (R).

Intervention		N	Mean	Std. Deviation
**EVGS Total (L)**	Barefoot	7	16.86	6.67
	AFO	11	14.00	4.47
	SMotO	11	8.55	4.43
**Total (L)**		29	12.62	5.94
**EVGS Total (R)**	Barefoot	7	17.14	6.77
	AFO	11	14.36	4.43
	SMotO	11	8.91	3.91
**Total (R)**		29	12.97	5.82

EVGS = Edinburgh Visual Gait Score; (L) = left; AFO = ankle-foot orthoses; SMotO = sensomotoric orthoses; (R) = right.

**Table 4:** Bonferroni comparison between the three orthoses.

	Intervention	Intervention	Significance
**EVGS** **Total (L)**	Barefoot	AFO	1.0
		SMotO	0.032 *
	AFO	Barefoot	1.0
		SMotO	0.027 *
**EVGS** **Total (R)**	Barefoot	AFO	1.0
		SMotO	0.052
	AFO	Barefoot	1.0
		SMotO	0.028 *

EVGS = Edinburgh Visual Gait Score; (L) = left; AFO = ankle-foot orthoses; SMotO = sensomotoric orthoses; (R) = right; * Indicates significance differences.

## Appendix A

**Table A1:** Participants with three scores (participant 1–7).

CHILD>	Participant 1					
Section	Score Barefoot		Score AFO		Score SMotO	
	Left	Right	Left	Right	Left	Right
Foot	12	12	7	7	5	7
Knee	7	7	5	5	3	3
Hip	2	3	2	3	2	2
Pelvis	2	2	2	2	1	1
Trunk	3	3	4	4	3	3
TOTAL	26	27	20	21	14	16
EVGS rating	severe	severe	moderate	moderate	Moderate	moderate
	Participant 2					
Section	Score Barefoot		Score AFO		Score SMotO	
	Left	Right	Left	Right	Left	Right
Foot	6	6	1	3	1	1
Knee	1	2	1	1	1	1
Hip	2	2	2	2	2	2
Pelvis	1	2	1	2	0	0
Trunk	1	1	1	1	0	0
TOTAL	11	13	6	9	4	4
EVGS rating	mild	moderate	mild	mild	Mild	mild
	Participant 3					
Section	Score Barefoot		Score AFO		Score SMotO	
	Left	Right	Left	Right	Left	Right
Foot	11	10	6	6	10	11
Knee	4	1	1	1	3	4
Hip	1	2	1	2	1	1
Pelvis	2	1	2	1	1	2
Trunk	2	2	2	2	2	2
TOTAL	20	16	12	12	17	10
EVGS rating	moderate	moderate	moderate	moderate	Moderate	mild
	Participant 4					
Section	Score Barefoot		Score AFO		Score SMotO	
	Left	Right	Left	Right	Left	Right
Foot	5	6	7	7	2	2
Knee	3	3	3	3	2	1
Hip	1	2	2	1	0	1
Pelvis	2	2	2	2	1	2
Trunk	0	2	2	2	2	2
TOTAL	11	15	16	15	7	8
EVGS rating	Mild	Moderate	Moderate	Moderate	Mild	Mild
	Participant 5					
Section	Score Barefoot		Score AFO		Score SMotO	
	Left	Right	Left	Right	Left	Right
Foot	6	5	5	4	2	2
Knee	4	1	3	3	2	2
Hip	1	1	2	2	1	1
Pelvis	1	1	1	1	2	2
Trunk	1	1	1	1	1	1
TOTAL	13	9	12	11	8	8
EVGS rating	Moderate	Mild	Moderate	Mild	Mild	Mild
	Participant 6					
Section	Score Barefoot		Score AFO		Score SMotO	
	Left	Right	Left	Right	Left	Right
Foot	12	13	5	6	3	5
Knee	5	5	4	5	0	1
Hip	4	4	2	2	1	1
Pelvis	0	0	0	0	0	0
Trunk	4	4	3	3	3	3
TOTAL	25	26	14	16	7	10
EVGS rating	Severe	Severe	Moderate	Moderate	Mild	Mild
	Participant 7					
Section	Score Barefoot		Score AFO		Score SMotO	
	Left	Right	Left	Right	Left	Right
Foot	6	7	4	4	1	3
Knee	2	4	2	2	2	2
Hip	2	2	0	0	0	0
Pelvis	1	0	0	1	1	0
Trunk	1	1	2	2	0	0
TOTAL	12	14	8	9	4	5
EVGS rating	Moderate	Moderate	Mild	Mild	Mild	Mild

**Table A2:** Participants with two scores: AFO and SMotO (participant 8–11).

CHILD>	Participant 8			
Section	Score AFO		Score SMotO	
	Left	Right	Left	Right
Foot	7	9	5	7
Knee	5	5	5	5
Hip	0	0	0	0
Pelvis	4	4	1	2
Trunk	2	2	0	0
TOTAL EVGS rating	18 Moderate	20 Moderate	11 Mild	14 Moderate
CHILD>	Participant 9			
Section	Score AFO		Score SMotO	
	Left	Right	Left	Right
Foot	6	7	7	7
Knee	5	4	2	2
Hip	2	2	0	0
Pelvis	4	4	2	2
Trunk	3	3	1	1
TOTAL EVGS rating	20 Moderate	20 Moderate	12 Moderate	12 Moderate
CHILD>	Participant 10			
Section	Score AFO		Score SMotO	
	Left	Right	Left	Right
Foot	7	7	3	3
Knee	3	3	2	2
Hip	3	3	1	1
Pelvis	0	0	0	0
Trunk	1	1	0	0
TOTAL EVGS rating	14 Moderate	14 Moderate	6 Mild	6 Mild
CHILD>	Participant 11			
Section	Score AFO		Score SMotO	
	Left	Right	Left	Right
Foot	6	4	1	2
Knee	3	2	1	1
Hip	2	2	1	1
Pelvis	0	0	0	0
Trunk	3	3	1	1
TOTAL EVGS rating	14 Moderate	11 Mild	4 Mild	5 Mild

**Table A3:** Segmental total.

Foot Totals			
Participant	Barefoot	AFO	SMotO
1	24	14	12
2	12	4	2
3	21	12	21
4	11	14	4
5	11	9	4
6	25	11	8
7	13	8	4
Total	117	72	55
Knee Totals			
Participant	Barefoot	AFO	SMotO
1	14	10	6
2	3	2	2
3	5	2	7
4	6	6	3
5	5	6	4
6	10	9	1
7	6	4	4
Total	49	39	27
Hip Totals			
Participant	Barefoot	AFO	SMotO
1	5	5	4
2	4	4	4
3	3	3	2
4	3	3	1
5	2	4	2
6	8	4	2
7	4	0	0
Total	29	23	15
Pelvis Totals			
Participant	Barefoot	AFO	SMotO
1	4	4	2
2	3	3	0
3	3	3	3
4	4	4	3
5	2	2	4
6	0	0	0
7	1	1	1
Total	17	17	13
Trunk Totals			
Participant	Barefoot	AFO	SMotO
1	6	8	6
2	2	2	0
3	4	4	4
4	2	4	4
5	2	2	2
6	8	6	6
7	2	4	0
Total	26	30	22
